# Lifestyle behaviours or socioeconomic characteristics? Gender differences in covariates of BMI in Hungary

**DOI:** 10.1002/osp4.316

**Published:** 2018-12-12

**Authors:** E. Jarosz

**Affiliations:** ^1^ Centre for Time Use Research, Department of Sociology University of Oxford (Oxford, United Kingdom) Oxford UK

**Keywords:** BMI, lifestyle behaviours, time‐use, gender

## Abstract

**Objective:**

Lifestyle behaviours are everyday activities that result from individual's values, knowledge, and norms shaped by broader cultural and socioeconomic context. These behaviours affect body weight as well as overall health and are influenced by a number of social characteristics. The aim of this paper was to examine the net effects of lifestyle behaviours and socioeconomic factors on body mass index (BMI), and how these differed by gender.

**Methods:**

This study used the 2009/2010 Hungarian Time Use Survey combining behavioural records, background information, and measures of self‐reported health and weight. The sample (*n* = 7765) was representative for the Hungarian population. Multivariate linear OLS regression models were employed to analyse the net effects of lifestyle and sociodemographic variables.

**Results:**

Daily behaviours were associated with BMI for women, but not for men, except for smoking. Meals frequency and duration of sleep had negative effects on female BMI, whereas duration of TV viewing had a positive effect. Occupational class was associated with male BMI, but not with female. The strong negative effect of smoking was significant for both genders.

**Conclusions:**

Lifestyle behaviours were linked with female BMI, with socioeconomic characteristics impacting on male BMI. These results suggest that a gender‐specific approach may be appropriate to address obesity issues in the Hungarian population.

## Introduction

An increased body mass index (BMI) is a known risk factor for developing cardiovascular disease and different cancers 1, 2. The relationship between lifestyle behaviours and an individual's weight is well‐established in social and health research 3. Health lifestyle theories argue that the propensity to adopt positive health behaviours is a result of the interplay between individual motivations and structural factors, such as gender or socioeconomic status 4, 5.

Lifestyle behaviours have been operationalized as daily activities resulting from individual values, orientations, knowledge, and norms defined by the broader cultural, social and economic context 5. An individual's life circumstances affect their possibilities or constraints to adopt certain lifestyle behaviours 4. Lifestyle behaviours in this theoretical framework are closely linked to sociological theories of symbolic distinction in which individual's choices regarding daily practices are regarded as determined by their social position 6.

There is a substantial overlap between the variables recognized as key health lifestyle behaviours, that is dietary habits, physical activity, smoking, and drinking alcohol 4, 7, and behaviours having a major impact on an individual's BMI, that is food choices and eating practices, physical activity, TV viewing, and sleep 8, 9, 10, 11. Health lifestyle theories argue that focusing on a single or a small subset of behaviours does not sufficiently reflect the diversity of the social forces behind them 5. Furthermore, different lifestyle behaviours are associated with one another 4, and some practices may facilitate or constrain the other 12, 13.

Among lifestyle behaviours dietary intake and eating behaviours, getting an adequate amount of sleep, being physically active, and managing stress were listed as ‘key weight management behaviors’ (10, 122). For each of these groups, there is a set of indicators that may be generated using time‐use diaries. Some information, such as dietary intake or stress management, is not available, but diaries allow collecting information on prevalence, frequency, duration, and timing of selected activities over the day, which provide relevant information for analysing lifestyle behaviours linked with obesity.

This study included (i) frequency of eating, (ii) having breakfast, and (iii) duration of food preparation as lifestyle practices related to diet and eating. It also analysed (iv) duration of sleep, (v) time spent being physically active, (vi) time spent watching TV, (vii) smoking, and (viii) alcohol use as other possible covariates of BMI.

Frequency of meals and having breakfast have both been inversely related to BMI 14, 15, 16, 17. Time spent on food preparation might be indicative of the quality of diet as food prepared at home was shown to have a better nutritional profile than food eaten out of home 9, 18. The quality of an individual's diet may also be affected by the duration of sleep 11, as sleep deprivation has been shown to be associated with food preferences, total energy intake, and metabolic processes 19, 20. Levels of daily physical activity have been inversely associated with individual weight, as well as with the risk of developing metabolic syndrome or diabetes 3, 21, 22. In contrast, leading a sedentary lifestyle is considered a risk factor for these diseases 21, 22. Time spent on physical activity together with the duration of TV watching was shown to be better predictors of BMI in children than their diet 23. TV viewing has also been associated with less healthy eating practices and weaker control over food intake 24. Lastly, smoking is known as an appetite suppressor 12, with women especially citing this effect of smoking as the primary reason for not trying to quit 13.

Lifestyle practices have been theorized to reflect sociodemographic differences 4, 5, 6. At the same time, there are substantial inequalities in BMI in developed countries, and the prevalence of obesity and overweight differs across population groups 25. People in lower social positions tend to have higher average BMI 26, though in many cases gender moderates this effect. The inverse association between socioeconomic status (SES) and BMI has been consistently reported for women, but not for men 27.

Men and women have different attitudes to health lifestyle practices 4, and social norms regarding body weight differ by gender 26, 28, 29. Women are more likely to submit to class norms which impacts their attitude toward diet and physical activity 26, 27, 28. For women, particularly in higher SES categories, being ‘thin’ is highly desirable 29. They are also more likely to make lifestyle changes to maintain or achieve their desired weight, often experiencing greater social pressure to do so 26, 28, 29.

To account for the effect of SES, I used standard indicators of individual's social status that is education, income, and occupation. These measures were shown to form different associations with individual's BMI as well as with health‐related behaviours.

In particular, an individual's educational attainment tends to be significantly and inversely related to BMI 25, 26, with better educated individuals being more careful about their food choices. Education was also shown to be the main variable explaining class differences in eating patterns, including meal frequency 30.

Occupational norms may dictate which body types are considered attractive or socially acceptable in a given work environment. Slimmer individuals tend to be favoured in white‐collar jobs 25, while workers with obesity experience more discrimination in professional occupations 31.

Income disadvantage has been liked with a higher risk of obesity. Wealthier individuals have better access to good quality food, health care services, and quality leisure time 32, and often follow different eating patterns than those in low income categories 30. The financial situation of the household is likely to affect the diet of all family members, including children and youth 33. Overall, daily hardships faced by individuals in lower class positions might make some health‐related practices appear unimportant, too far‐sighted, or irrelevant compared to the more pressing demands of everyday life 34. Simple and inexpensive pleasures such as eating in fast food outlets or watching TV offer immediate gratification and as such, can be used as a means to alleviate stress 35.

There are few datasets that allow exploring the relationship between lifestyle practices, individual's SES and BMI using a detailed log of daily activities. Such analyses have been conducted on the American Time Use Survey (ATUS), but overall research on the topic is limited, particularly for large samples. In Europe, data including time‐use log as well as information on BMI was collected in two past‐2000 national Time Use Surveys: in Finland and Hungary. This study uses detailed behavioural accounts from the 2009/2010 Hungarian Time‐Use Survey.

The Hungarian context is interesting for obesity research as Hungary has the highest share of obese individuals in Europe, and the fourth highest among all OECD countries 36. It is also very unequal in terms of rates of overweight and obesity across population groups 25. Following a wide‐range evaluation of nutritional status of Hungarians, obesity has been recognized as a major public health threat 37.

Lifestyle behaviours are a result of the interplay between individual preferences and social, economic, and cultural factors, including gender norms, knowledge about nutrition, or financial constraints. Some of these behaviours, such as eating patterns, physical activity, or sleep, are related to an individual's weight status and obesity risk, while individual SES indicators form independent associations with BMI. The objective of this paper was to disaggregate the effects of lifestyle behaviours and individual socioeconomic characteristics on BMI. It was hypothesized that men and women would differ in terms of the effect of their lifestyle behaviours on BMI, net of their socioeconomic characteristics. As women are more likely to adopt a positive health lifestyle and overall experience greater normative pressure regarding their weight, lifestyle behaviours were expected to have greater effect on female BMI than on male.

## Methods

This study used detailed behavioural records from the most recent Hungarian Time Use Survey (HTUS). HTUS is a nationally representative survey which collected data between October 2009 and the end of September 2010. Time‐use diaries provide highly accurate and reliable estimates of an individual's time allocation 38. It is very rare for time‐use surveys to collect data on an individual's height and weight but there are few exceptions: American, Finish, and Hungarian survey include information on self‐reported height and weight.

HTUS provided time‐use records for 8391 individuals (one day per person) aged 10 to 84. The following study uses a subsample of diarists aged 18 and above (*n* = 7765). Younger respondents were excluded due to the low number of cases and the fact that there are issues with BMI estimates for children and adolescents 39. Women accounted for 53% of the sample selected for analyses. The mean age of respondents was 44 years ±18.5 standard deviation (SD). More detailed information regarding the sociodemographic characteristics of the sample is given in the Appendix.

Time spent in selected activities, their incidence, and frequency were computed based on the respondent's time diaries. Each diary recorded primary (main) and secondary (additional) activities over a 24‐hour period. Estimates analysed in this study used combined data from both the primary and secondary activities sequences. The duration of an activity (food preparation, sleeping, TV viewing, physical activity) was computed based on the total duration of all episodes of that activity occurring throughout the day, regardless of whether it was recorded in the primary or secondary activity sequence. In this study, time spent on physical activity included any intentional exercise as well as walking, or active travel, which is a more relevant depiction of daily activity levels than exercise alone. The number of meals is equal to the number of all episodes of eating, and the incidence of breakfast was computed based on whether a respondent reported eating breakfast, as there was a separate code for each type of meal. The incidence of smoking or alcohol use were based on whether an individual reported any episode of smoking or drinking alcohol over the 24 hours.

Regarding socioeconomic characteristics, the original Hungarian occupational category FEOR‐08 was recoded into categories corresponding to the 1‐digit ISCO codes. It included the following groups: (1) managers and professionals, (2) technicians and associated professionals, (3) clerical occupations, (4) sales and service occupations, (5) agriculture, fishing and forestry jobs, (6) craftsmen, industry, trade and construction occupations, (7) machine operators, and (8) low‐skill jobs. The last category (9) included missing values which corresponded to being out of the labor market. Codes for army jobs were dropped due to the low number of observations.

Individual monthly income was originally given in Hungarian Forint (1000 Ft ≈ 3 EUR). The income variable for the study was generated by collapsing original ten income bands into four broader income groups: (1) under 80000 Ft (Hungarian Forint), (2) between 80001 and 160000 Ft, (3) between 160001 and 300000 Ft, (4) 300001 to 1000000 Ft.

Education was constructed based on the original variable corresponding to the highest completed level of education and was divided into the following categories: (1) incomplete primary (including no education), (2) complete primary, (3) vocational, (4) secondary, incomplete or complete, (3) tertiary.

Models introducing sociodemographic factors also included marital status and type of settlement as additional control variables. Marital status included the following categories: (1) single, (2) married or cohabiting, (3) divorced or widowed. The type of settlement differentiated between living in (1) a county city, including Budapest, (2) a town, and (3) a village/rural settlement.

All models controlled for age and self‐reported health. Age was continuous, whereas self‐reported health consisted of the following categories: (1) very good, (2) good, (3) satisfactory, (4) bad, and (5) very bad.

At the first stage of analyses, descriptive statistics were produced to illustrate sociodemographic differences in mean BMI values in Hungary. The paper presents the distribution of BMI categories by gender, which indicate how many respondents would qualify as being underweight (BMI < 18.5), having normal weight (18.5–24.9), being overweight (25–29.9) or obese (BMI ≥ 30). Next, the results show mean BMI values by gender and by education, occupation, and income category, then gender differences in time allocation to selected activities are described.

The main stage of analyses involved running a set of multivariate OLS regression models separately for men and women. The first model presents associations between individual BMI and selected lifestyle behaviours: frequency of eating, having breakfast on the diary day, time spent on food preparation (in hours), use of alcohol or tobacco, time spent on physical exercise (in hours), duration of sleep (in hours), and time spent in TV watching, also in hours. Age and health status were included as control variables. The second model used all variables from the first model, adding indicators of an individual's socioeconomic characteristics, that is education, income, occupational category, and additional control variables: marital status, and type of settlement where respondent lived. Missing values for structural variables were included in the model to maintain the same sample size as in the case of the model analysing only behavioural variables.

## Results

**Figure 1:**
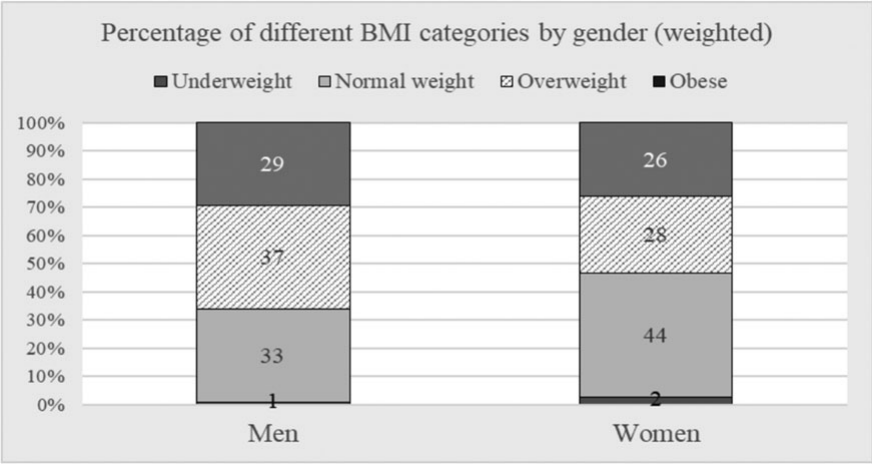
Figures 1 and 2 contains poor quality of text. Please resupply if necessary.BMI categories, by gender, weighted.

Around 54% of women and 66% of men in the Hungarian sample were classified as individuals being overweight or obese (Figure 1). The mean BMI for women was 26.0 ± 5.2 SD and for men was 26.9 ± 4.7 SD.

The mean BMI levels differed with regard to an individual's socio‐economic characteristics (Table 1), that is, better educated or more affluent women had a significantly lower BMI. In both cases the relationship with BMI was linear. Women with tertiary education had the lowest mean BMI at 24.6 (*p* < 0.01), as did women with the highest earnings with a BMI of 24.7 (*p* < 0.05).

**Table 1:** Mean BMI values by individual's structural categories, weighted.

	**Male**		**Female**	
	Mean	95% CI	Mean	95% CI
Education				
Incomplete primary	26.6	(25.7–27.5)	27.6	(26.9–28.3)
Completed primary	26.8	(26.4–27.3)	26.7	(26.4–27.1)
Vocational	27.3	(27.0–27.5)	26.2	(25.9–26.5)
Secondary	26.3	(26.0–26.6)	25.2	(24.9–25.5)
Tertiary	26.6	(26.2–26.9)	24.6	(24.3–24.9)
Individual income				
<80000 Ft	26.4	(25.8–27.0)	25.8	(25.2–26.4)
80001–160000	27.0	(26.7–27.3)	25.7	(25.4–26.0)
160001–300000	26.7	(26.4–27.0)	25.1	(24.8–25.4)
300001–1000000	25.5	(24.9–26.1)	24.7	(24.0–25.4)
Missing	27.0	(26.7–27.3)	26.2	(25.0–26.5)
Occupation				
Managers and professionals	26.6	(26.3–26.9)	25.3	(25.0–25.7)
Technicians	26.9	(26.4–27.4)	25.4	(25.1–25.8)
Clerks	26.4	(24.4–28.4)	25.8	(25.3–26.2)
Sales and services	27.0	(26.5–27.5)	25.8	(25.5–26.3)
Agriculture	27.2	(26.5–28.0)	27.9	(26.9–28.8)
Trade, industry, construction	27.5	(27.2–27.8)	26.5	(25.9–27.0)
Machine operators	27.7	(27.2–28.1)	26.7	(26.2–27.3)
Low skill jobs	26.4	(25.8–26.9)	26.8	(26.4–27.2)
Missing (not in employment)	23.5	(23.0–24.0)	23.5	(23.0–24.0)

For men, the association between income and BMI was curvilinear, with the wealthiest and poorest men having the lowest mean BMI (25.5 and 26.4, respectively), but only the difference between the men with the highest income and the medium income categories was significant (*p* < 0.05). Male BMI was not significantly differentiated by educational attainment, except for the statistically significant, but not substantial difference, between men with vocational education and men with secondary or tertiary education (p < 0.05).

Regarding occupational category, women in white‐collar jobs (managers and professionals, technicians, and clerical positions) had a mean BMI below 26.0, while women in agriculture, blue‐collar jobs (industry and construction workers, machine operators) and low‐skill jobs had a mean BMI above 26.0. The difference between women in managerial and professional occupations and women in blue‐collar jobs, agriculture, or low‐skill jobs was significant (*p* < 0.05).

Occupational class also impacted on male BMI, with men in the highest occupational positions (managers and professionals) and lowest occupations (unskilled labor force) having the lowest BMI at 26.6, and 26.4, respectively. These values were significantly lower than men in sales and service occupations, agriculture, industry, and machine operators (*p* < 0.05).

**Figure 2:**
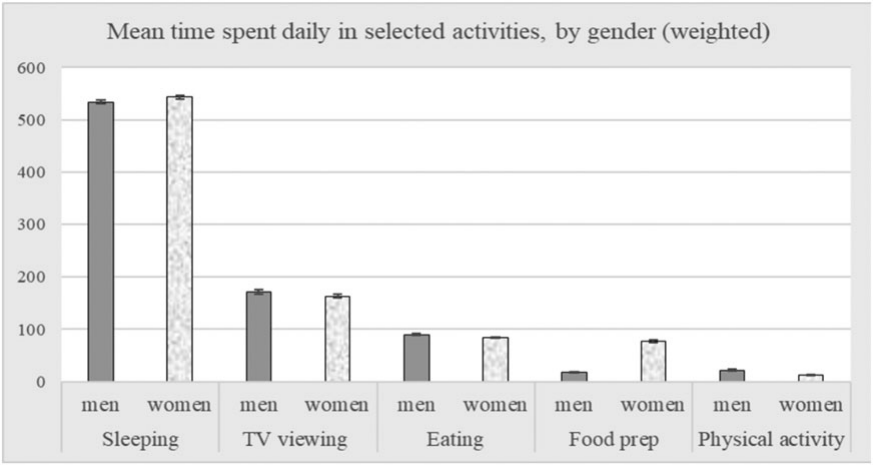
Mean time (95% CI) in minutes spent in selected activities, by gender, weighted.

Men and women differed with regard to how much time they spent on selected daily activities (Figure 2). Women dedicated significantly less time to eating than men (84 minutes versus 90 minutes; *p* < 0.01), which translated to having fewer meals per day, though in this case, the difference was minor and not statistically significant (2.96 and 2.99 meals respectively), implying that women had nearly as many meals as men, but they were of shorter average duration. Women spent substantially more time (4 times longer; *p* < 0.01) on food preparation, while men spent more time being physically active (almost 10 minutes more per day; *p* < 0.01) and watching TV (9 minutes more; p < 0.01).

Associations between individual lifestyle behaviours and BMI are presented in Model 1 (Table 2). In case of women, most analysed behaviours were associated with BMI. The number of meals was negatively associated with BMI (*p* < 0.05), as well as duration of sleep (p < 0.01), and time spent being physically active (p < 0.05). In contrast, the duration of food preparation and time spent on TV viewing were positively associated with female BMI (*p* < 0.001 for both coefficients). Lastly, smoking had the strongest negative effect on female BMI, with smokers having nearly 1‐point lower BMI compared to non‐smokers (p < 0.001). Smoking was also the only activity that had a significant effect on male BMI, which was on average lower by 0.8 among men who smoked (p < 0.001).

**Table 2:** Behavioural and structural covariates of BMI, by gender.

	**MODEL 1**		**MODEL 2**	
	Male	Female	Male	Female
Age	0.059***(0.01)	0.063***(0.01)	0.030***(0.01)	0.052***(0.01)
Self‐reported health *(ref. very good)*				
Good	0.768***(0.23)	0.609*(0.27)	0.460* (0.23)	0.291 (0.27)
Satisfactory	1.350***(0.26)	1.826***(0.29)	1.023***(0.26)	1.419***(0.29)
Bad	1.042**(0.35)	2.506***(0.34)	0.815* (0.35)	1.987***(0.34)
Very bad	0.308 (0.49)	2.602***(0.48)	0.109 (0.49)	1.993***(0.48)
Number of meals	−0.188 (0.13)	−0.251*(0.12)	−0.185 (0.12)	−0.299* (0.12)
Ate breakfast *(ref. did not eat)*	−0.190 (0.25)	0.150 (0.24)	−0.245 (0.24)	0.193 (0.24)
Duration: food preparation	−0.199 (0.12)	0.251***(0.06)	−0.099 (0.12)	0.091 (0.06)
Consumed alcohol *(ref. did not consume)*	−0.370 (0.27)	−0.010 (0.66)	−0.322 (0.27)	0.274 (0.66)
Smoked cigarettes *(ref. did not smoke)*	−0.792***(0.25)	−0.977***(0.30)	−0.949***(0.25)	−1.040*** (0.30)
Duration: physical activity	−0.100 (0.09)	−0.252*(0.13)	−0.058 (0.09)	−0.150 (0.13)
Duration: TV	0.029 (0.04)	0.146***(0.04)	0.039 (0.04)	0.167*** (0.04)
Duration: sleep	−0.042 (0.04)	−0.104**(0.04)	0.011 (0.04)	−0.078* (0.04)
Individual income *(ref. <80000 Ft)*				
80001–160000			0.479 (0.36)	0.497 (0.30)
160001–300000			0.434 (0.37)	0.452 (0.32)
300001–1000000			−0.166 (0.51)	0.591 (0.51)
Missing			0.433 (0.35)	0.679* (0.29)
Education *(ref. <incomplete primary)*				
Complete primary			0.967* (0.49)	0.278 (0.38)
Vocational			0.659 (0.48)	0.275 (0.41)
Secondary			0.518 (0.50)	0.198 (0.42)
Tertiary			0.644 (0.55)	−0.519 (0.48)
Occupation *(ref. managers and professionals)*				
Technicians			0.274 (0.34)	−0.269 (0.29)
Clerks			0.427 (0.94)	−0.387 (0.33)
Sales and services			0.613 (0.35)	0.035 (0.33)
Agriculture			0.521 (0.46)	0.700 (0.51)
Trade, industry, construction			0.769* (0.31)	−0.063 (0.38)
Machine operators			0.949** (0.34)	0.369 (0.39)
Low skill jobs			−0.256 (0.40)	0.031 (0.36)
Missing			−1.444***(0.42)	−0.500 (0.40)
Marital status *(ref. married)*				
Single			−1.190***(0.23)	−1.589***(0.26)
Widowed/divorced			−0.968***(0.24)	−0.557**(0.19)
Settlement type *(ref. Budapest/county city)*				
Other city/town			0.291 (0.19)	0.288 (0.18)
Village			0.247 (0.20)	0.490* (0.20)
Intercept	24.522***(0.51)	22.400***(0.52)	24.575***(0.89)	23.033***(0.87)
R‐squared/N	0.08/3445	0.13/4320	0.11/3445	0.15/4320

* denotes sig level < =0.05

** denotes sig level < =0.01

*** denotes sig level < =0.001

The greater significance of daily activities for female BMI was also reflected in the percentage of variance explained by the model. In case of men, the R‐squared value for the model was 8%, and 13% for women. There was also a significant association between BMI and the control variables, age and self‐reported health for both genders.

Model 2 added the individual's socioeconomic characteristics education, occupation, income, and additional control variables, marital status and type of settlement (Table 2). In the case of women, the coefficients for duration of food preparation and physical activity became insignificant, which means the effects of these behaviours were explained by socioeconomic factors. The number of meals was significantly and inversely associated with BMI (*p* < 0.05), with every additional meal linked to a reduction in BMI by, on average, 0.3 points. The effect of sleep remained significant and negative though slightly weaker (p < 0.05). Every additional hour spent sleeping was associated with a BMI lower by approximately 0.1 point. The effect of TV viewing was positive, with every additional hour spent on TV viewing linked to an increase in BMI by approximately 0.2 point (*p* < 0.001). The incidence of smoking had a negative effect on BMI and was stronger than in Model 1. Women who smoked had a BMI lower by approximately 1 point compared to those who did not smoke (*p* < 0.001). Married/cohabiting or divorced/widowed women had a higher BMI than women who were single (p < 0.001 and *p* < 0.01, respectively). Lastly, women living in rural areas had a higher BMI than those living in the cities (*p* < 0.05).

As in Model 1, smoking was the only behavior associated with BMI for men. Men who smoked had on average nearly 1‐point lower BMI compared to those who did not smoke (*p* < 0.001). Occupational characteristics in Model 2 were linked with male BMI but not with female. Men working in trade/industry or construction jobs, or as machine operators (all of which are blue‐collar jobs) had a significantly higher BMI (p < 0.05 and *p* < 0.01, respectively), with men who did not work having a BMI lower by over 1‐point (*p* < 0.001). Lastly, married or divorced men had a higher mean BMI than their single counterparts (p < 0.001 for both coefficients).

## Discussion

This study provided empirical evidence of the association between everyday lifestyle behaviours and an individual's BMI, demonstrating that there are substantial gender differences in this dimension. In line with the hypothesis, the effect of lifestyle practices on BMI was stronger for women than for men. Furthermore, the effects of several types of daily behaviours on female BMI (frequency of meals, duration of sleep and duration of TV viewing) were also significant when socioeconomic factors were accounted for.

The effects of time spent on food preparation and time spent being physically active were significant for female BMI only in Model 1 which did not control for socioeconomic characteristics. The longer time spent in food preparation, indicative of the fact that the food was prepared at home, was associated with a significantly higher BMI. This finding might seem contradictory to the fact that home‐made food tends to have a better nutritional profile and lower fat content than food eaten out of home. However, this result is consistent with earlier findings that link longer time spent in food preparation with a higher female BMI, showing that time in food preparation is a moderator between BMI and a greatest interest in cooking 28. It is possible that women who spend more time preparing food at home are more interested in culinary practices, which has been linked with higher body weight 28; however, this could not be tested with the available data.

The fact that the effects of physical activity and duration of food preparation on female BMI were fully accounted for by an individual's socioeconomic characteristics reveals possible reasons behind SES‐related health inequalities existing among Hungarian women. Specifically, the time spent in physical activity and time dedicated to preparing food at home are not distributed equally across the population, which is important since Hungary is a country with very high absolute socioeconomic inequalities in weight status 25.

Out of all the behaviours analysed in this study, only smoking was significantly associated with male BMI. The negative effect of smoking was strong and significant across all models for both men and women. Healthy lifestyle choices, such as preparing one's food or exercising (in case of men), did not necessarily mean that one would have a lower, or ‘healthier’ BMI, whereas the unhealthy practice of smoking has been shown to be the strongest predictor of lower BMI for both men and women, regardless of their socioeconomic characteristics. Firstly, it shows that practices associated with a positive health lifestyle and those linked with having lower BMI may diverge. Secondly, it implies that policy makers might need to address fears of weight gain among current smokers to increase the effectiveness of anti‐smoking campaigns.

Men and women also differed with regard to the structural covariates of BMI. Specifically, in Model 2, occupational characteristics were associated with male BMI, but not with female. Men in blue‐collar jobs had nearly a 1‐point higher BMI than their counterparts in managerial and professional occupations. This result is likely to reflect the fact that automatization of industrial processes eliminated some opportunities for physical exertion among those occupational groups 40, while dietary practices remained greatly differentiated by an individual's occupational class 41.

This study had several limitations. Firstly, it did not include information on what a person ate on the diary day as this information was not available. While eating patterns have been shown to be linked with individual weight, including dietary information might provide further insight on some of the findings, such as the positive association between the longer time spent on food preparation and higher BMI for women. Secondly, limitations of the diary as an instrument might have affected the data on smoking or alcohol consumption. These measures are likely to be underestimated due to the short duration of these activities (smoking in particular), which may make respondents consider them too short to be reported. Heavy smokers or heavy drinkers were more likely to report these activities as in their case, there are more (and possibly longer) episodes of smoking or drinking in the sequence. Furthermore, smoking and alcohol use are viewed as socially undesirable in some social groups and they may have been underreported. Lastly, some of the activities, such as drinking alcohol or sport participation (a component of physical activity variable), may not happen daily, but be done several times per week. While it is likely that the overall participation rates captured by the diaries are exact at the population level, in terms of individual BMI outcomes, having weekly estimates of such activities might be more informative.

The study findings suggest that different policy measures may need to be adopted to address obesity in different population groups. Consequently, the same regulations might have different effects depending on who they are aimed at. In general, there is no one‐fits‐all solution, which is something that policymakers in Hungary seem to ignore. In 2011, a tax on fatty and sugary foods was introduced, clearly a measure targeted at changing individual behaviours at the population level. This triggered equity concerns because such foods are generally more likely to be purchased by lower‐income individuals. Although the tax proved effective in lowering consumption of fatty foods and sugary drinks 42, it is very difficult to measure whether and how it affected existing inequalities in health and weight. The effectiveness of the tax regarding addressing obesity issues in Hungary is therefore questionable.

As this research demonstrated, numerous daily practices were linked with female BMI, and occupational class was associated with male BMI. This finding suggests that a policy targeted at changing daily habits might be more effective for women, while a structural approach and occupation‐based interventions might be more relevant for men. This may involve examination of men's dietary and eating habits versus their activity levels (measured using metabolic equivalents, or METS scores) during working time in those occupational categories for which the highest average BMI values were reported. As occupational characteristics shape leisure time behaviours, activities undertaken during the out‐of‐work hours may also be examined. Time‐use diaries were shown to be a reliable source of data for this purpose 43, and this is also a possible direction toward which the present study can be expanded.

## Funding

This research was supported by the Economic and Social Research Council (ESRC) grant number ES/L011662/1.

## Conflict of interest

The author declared no conflict of interest.

## Appendix A

**Table 1:** Sample structure

Education	N	Percent
Incomplete primary or none	624	7%
Completed primary	1827	22%
Vocational	2171	26%
Secondary	2360	28%
Tertiary or equivalent	1409	17%

Income	N	Percent
80000 or less	584	7%
80001–160000	2252	27%
160001–300000	2072	25%
300001–1000000	301	4%
Missing	3182	38%

Occupation	N	Percent
Legislators, managers and professionals	1291	15%
Technicians & associate professionals	1008	12%
Clerical occupations	450	5%
Sales & services workers	1009	12%
Agriculture, forestry workers & fishermen	308	4%
Trade, industry, construction workers	1323	16%
Machine operators	854	10%
Unskilled labour force	921	11%
Missing (unemployed, student, retired)	1227	15%

Marital status	N	Percent
Single	2366	28%
Married or cohabiting	4110	49%
Widowed or divorced	1915	23%

Settlement type	N	Percent
Capital/county city	2807	33%
Town	2956	35%
Village	2628	31%

Health status	N	Percent
Very good	1051	13%
Good	2846	34%
Satisfactory	2776	33%
Bad	981	12%
Very bad	283	3%
Missing	454	5%
